# Designing a minimum data set of laboratory data for the electronic summary sheet of pediatric ward in Iran: A cross‐sectional study

**DOI:** 10.1002/hsr2.1315

**Published:** 2023-06-07

**Authors:** Farzaneh Shahbakhsh, Reza Khajouei, Azam Sabahi, Yousef Mehdipour, Leila Ahmadian

**Affiliations:** ^1^ MSc of Health Information Technology, Vice Chancellor for Treatment Affairs Zahedan University of Medical Sciences Zahedan Iran; ^2^ Department of Health Information Sciences, Faculty of Management and Medical Information Sciences Kerman University of Medical Sciences Kerman Iran; ^3^ Department of Health Information Technology, Ferdows School of Health and Allied Medical Sciences Birjand University of Medical Sciences Birjand Iran; ^4^ Paramedical School Torbat Heydariyeh University of Medical Sciences Torbat Heydariyeh Iran

**Keywords:** clinical laboratory information systems, electronic health records, hospital information systems, laboratory data, minimum data set

## Abstract

**Background and Aim:**

Iranian hospitals are provided with hospital information systems (HISs) from different vendors, which make it hardly possible to summarize laboratory data in an consistent manner. Therefore, there is a need to design a minimum data set of laboratory data that will define standard criteria and reduce potential medical errors. The purpose of this study was to design a minimum data set (MDS) of laboratory data for an electronic summary sheet to be used in the pediatric ward of Iranian hospitals.

**Methods:**

This study consists of three phases. In the first phase, out of 3997 medical records from the pediatric ward, 604 summary sheets were chosen as sample. The laboratory data of these sheets were examined and the recorded tests were categorized. In the second phase, based on the types of diagnosis we developed a list of tests. Then we asked the physicians of the ward to select which ones should be documented for each patient's diagnosis. In the third phase, the tests that were reported in 21%–80% of the records, and were verified by the same percentage of physicians, were evaluated by the experts' panel.

**Results:**

In the first phase, 10,224 laboratory data were extracted. Of these, 144 data elements reported in more than 80% of the records, and more than 80% of experts approved them to be included in the MDS for patients' summary sheet. After data elements were investigated in the experts' panel, 292 items were chosen for the final list of the data set.

**Conclusions:**

This MDS was designed such that, if implemented in hospital information systems, it could automatically enable registering data in the summary sheet when patient's diagnosis is registered.

## 1 INTRODUCTION

A minimum data set (MDS) is typically designed at national and regional levels with the purpose to improve data interchange and comparability in these scales. Using an MDS, one can standardize the information system's content.^1^ MDS is used for policy‐making, planning, and decision‐making, and it plays a major role as a health care indicator at the national level within different institutions.^2^ A standard minimum data set by improving documentation of the medical records can improve care and reduce complaints. It also allows physicians to decide on clinical care and workflow management as well as quality activities.^3^

The design of a MDS should follow realistic and feasible criteria. However, in the clinical field, due to the lack of a specific measurement scale, implementing this objective has its fair share of barriers and challenges.^4^ One of the important factors in developing an MDS is that users must understand the importance of applying it and have the necessary knowledge in this regard.^5^ Furthermore, designing an MDS requires a well‐defined framework that covers the information needs of its intended users.^6^ Studies have shown that designing a minimum data set helps define standard criteria that can lead to the reduction of medical errors.^7^ It enhances data collection and facilitates prediction of illnesses and prevents their outbreak Moreover, it can be a strong tool for collecting data to be used at national and regional levels.^8^ Besides, it gives rise to a better working relationship between care providers, which in turn improves their performance within the hospitals.^9^ It enables information gathered during a hospitalization to be shared with other health care professionals outside the hospital, which can help complete the medical record of the patient in the future.^10^

When completing a summary sheet, either in an electronic‐based medical record system or in a manual system, it is necessary to register a summary of the most important patient's clinical findings. Today, some electronic health records systems are easily able to summarize medical data and save the time of care providers.^11^ Various studies have indicated that utilizing an electronic summary sheet (ESS), even if it requires manual data entry by health care providers and does not pull the data automatically from the hospital database, is desirable among care providers and improves the completeness and quality of documentation.^12^, ^13^ Experts believe summary sheets that are created automatically, compared to those that are created manually or dictated, are more likely to be filled out more quickly and are more acceptable for health care providers in both outpatient and inpatient departments as a result.^14^

Iranian hospitals have Hospital information systems (HISs) from multiple different vendors. Many of these system are not able to summarize laboratory data and prepare an automatic and electronic summary sheet.^15^ The HISs in teaching hospitals in Zahedan have been developed by a commercial company. A summary sheet for pediatric patients has been designed within this system, but the results of laboratory tests in this sheet still need to be registered by physicians due to a deficiency in that sheet's design (i.e., lack of a MDS of laboratory data).^16^ This has led to a widespread dissatisfaction among physicians working in these hospitals.^17^ The reason for this is the wide variation in the different types of laboratory tests and the fact that the type of tests performed depend on the diagnosis of patients.

It should be noted that so far, to the best of our knowledge, no MDS of laboratory data has been designed for a summary sheet in Iran. In addition, the attending pediatricians of these centers have expressed that the such a MDS of laboratory results can help them teach medical students. Therefore, designing a minimum data set of laboratory data for an electronic summary sheet for use in the pediatric department can enable the automatic transfer of patients' laboratory data to their own summary sheet.

## 2 MATERIALS AND METHODS

This is a cross‐sectional descriptive study conducted in three phases in Zahedan. In the first phase, we examined the summary sheets of patients admitted to the pediatric ward of Ali ibn Abi Talib Hospital between 2015 and 2016. As a results an initial list of laboratory data to be used as the input for the next two phases was prepared. (Zahedan, has two teaching hospitals that provide care for children: Ali Ibn Abi Talib‐ a general hospital that has a pediatric ward‐ and the Ali Asghar hospita‐ a specialized pediatric hospital that is the only hospital in the city that provides short‐term care for children). We employed an integrated approach, consisting of examining available documentations and consulting expert opinionsto develop the MDS.

### 2.1 Phase 1: Review of summary sheets

The number of patients admitted to the pediatric ward of Ali ibn Abi Talib Hospital was 3997 during the course of 1 year. From this population, a sample of 350 summary sheets of patients were chosen using Cochran's sample size formula. To select the patient record randomly, an average of 29 patient records per month and one record per day were extracted from the hospital information system. Then, we examined the summary sheets of these 350 patients' records, and the laboratory data reported in their summary sheets were extracted. Once we extracted the data from the medical record we categorized our data using diagnosis codes according to ICD‐10 (International Statistical Classification of Diseases and Related Health Problems) chapters (Supporting Information: See Appendix 1). Then, purposive sampling was used in a stepwise manner to avoid overlooking any laboratory data related to specific and less common diagnoses. After the initial random sampling, we selected additional 10 records for diagnoses that were coded in a single chapter of the ICD‐10 system with the intent to provide more extensive and unbiased data on conditions that are less common and might have been otherwise overlooked with our original method. At this stage, we reviewed our data and if no new laboratory data appeared to the previously extracted list, sampling was completed for the diagnoses of those chapters. Otherwise, sampling continued until no new laboratory data was added to the list after reviewing 10 additional summary sheets.

A data extraction form was developed to collect the required data from the summary sheets. Using this form, in addition to laboratory data, other data such as patient's record number, age, sex, and final diagnosis were extracted from the medical records. The validity of the data collection form was approved by three experts who specialize in medical informatics and health information technology (HIT). Depending on the diagnosis, there was significant variability in the laboratory data summarized in the summary sheets. To simplify the presentation and organization of the findings, all final diagnoses were coded based on the ICD‐10 system. Coding was done by one of the researchers and the outcome was checked by a HIT expert. Data were analyzed based on their frequency and categorized into four groups: uncommon (0%–25%), less common (26%–50%), common (51%–75%), quite common (76%–100%) (Supporting Information: See Appendix 2). In that the required laboratory data have been specified based on each distinct chapter ICD10.

### 2.2 Phase 2: Physicians perspective

In this phase, pediatricians of the ward were surveyed regarding the laboratory tests that should be recorded in the summary sheet. To determine their viewpoints, a checklist including laboratory tests was developed based on the results of the first phase. In this list, the laboratory data were categorized based on the classification of diagnoses by the ICD‐10 chapters. The pediatricians determined the necessity of the reporting of the laboratory tests results in the patient's summary sheet in this list. The study population consisted of 23 pediatrics specialists and subspecialists working in the two abovementioned teaching hospitals. All pediatricians working in these hospitals were invited to participate in the study, and 18 specialists accepted the invitation. The pediatricians proceeded based on their own medical judgment concerning the necessity of including/excluding specific data elements. The validity of the developed list was approved by three experts who specialize in the field of medical informatics and health information management with at least 5 years of working experience.

### 2.3 Phase 3: Experts' panel

**Figure 1:**
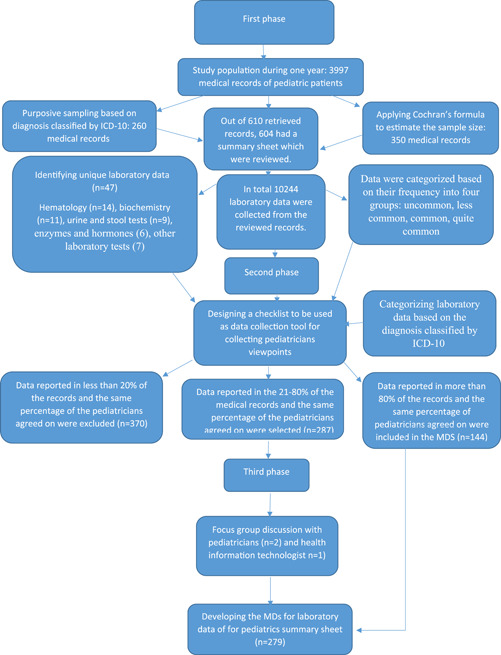
Study steps.

In the third phase, an MDS of laboratory tests for the pediatric summary sheet was designed in the experts' panel (including pediatricians and health information management specialists with 5 years of working experience) based on the results of the first and second phases. Specifically, the laboratory data reported in more than 80% of the sheets in the first phase and verified by more than 80% of physicians in the second phase were considered necessary data and were consequently included in the MDS. On the other hand, data mentioned in less than 20% of summary sheets and confirmed by 20% of physicians were excluded from the final draft of the MDS. In this phase, discussions were made by the experts' panel on data that was reported in 21%–80% of summary sheets or approved by the same percentage of physicians in the second phase. For this purpose, a list of these data was provided. The validity of the list was approved by three specialists in the field of medical informatics and health information management. The list was reviewed by the experts' panel in order to verify or reject the inclusion of each laboratory data in the final list of MDS. The study process are presented in Figure 1.

The discussion continued until a final agreement was reached on removing or adding any data to the final MDS list.

The experts' identities and responses were kept confidential during the design of the laboratory minimum data set. Moreover, their participation in the validation stages was voluntary, and they were free to withdraw from the study at any stage.

Data analysis was done using SPSS 16, including descriptive statistics (frequency and percentage). Eventually, the results of the three phases of the study were combined.

## 3 RESULTS

In this study, 610 summary sheets of patients admitted to the pediatric ward of Ali ibn Abi Talib Hospital, Zahedan, were explored. Of the 350 extracted patients' records at the first stage, 6 did not have a summary sheet and were thus discarded. In the purposive sampling stage, 260 summary sheets were retrieved. Totally, 604 patient summary sheets were investigated. In tota, 58 different diagnoses wad coded by ICD‐10. The recorded gender for almost half of the patients (*n* = 316, 52%) was male. The age range of the patients was between 10 days and 11‐year‐old.

Of the 21 chapters of ICD‐10, Chapter 15, which includes obstetric and childbirth diseases, was not used in coding because it is not applicable in coding pediatric diagnosis. Similarly, Chapter 20, which is related to external causes of injury, was not used as the nature of injuries encoded using Chapter 19. In addition, during the 1‐year period of the study no case of eye diseases admitted to the pediatric ward of the studied hospital. This was due to the fact that these patients could have been admitted to the local ophthalmic hospital. Besides, during the same period, no patient with ear diseases was hospitalized in this ward. Therefore, to incorporate the laboratory tests related to these diagnoses into the MDS, in the third phase of the study, the expert panel agreed to include the laboratory tests that were reported in more than 80% of summary sheets of other records necessary data elements for Chapters 7 and 8 (Supporting Information: See Appendix 3). Thus, out of the 21 chapters of ICD‐10, 17 chapters were used in the first phase to classify data. A total of 10,224 laboratory data were extracted from the summary sheets, with 47 unique data elements. Among these unique elements, 14 data elements were related to hematology, 11 to blood biochemistry, 9 to urine and stool analysis, 6 to enzymes and hormones, and 7 to other laboratory tests.

The laboratory data that were reported in less than 20% (370) of summary sheets were removed from further evaluation. Then, we prepared a list of 801 data elements, classified based on the diagnoses coded by different chapters of ICD‐10. A total of 144 data elements were reported in more than 80% of summary sheets, and more than 80% of the physicians approved them in the second phase, thereforeconsidered necessary elements. Hence, they were added to the laboratory MDS. The remaining 287 data elements were examined in the third phase by the expert panel. Of these, 148 data elements were considered essential data and included in the MDS. Ultimately, a total of 292 data elements were incorporated in the MDS of laboratory tests for the ESS.

Table 1 shows 238 data elements (14 unique laboratory tests, * 17 chapters of ICD10 = 238 data) of hematology tests reported in the summary sheets based on the diagnosis classification made by ICD‐10 chapters. Of these tests, 67 data elements were reported in more than 80% of the summary sheets. About these data elements 80% of specialists were in agremment about their inclusion in the MDS. On the other hand, 67 data elements were reported in less than 20% of the sheets and were consequently excluded from the study. Of the 104 remaining data elements, 49 were considered necessary by the experts panel. Finally, a total of 116 data elements related to hematology tests were listed in the MDS.

**Table 1:** The frequency of hematology tests reported in patient summary sheets, verified by physicians and included in MDS.

14 data	WBC			WBC/LY			WBC/other			Hb			PLT			RBC			HCT			MCV			MCH			MCHC			PT			PTT			INR			BC		
ICD‐10 chapter	1	2	3	1	2	3	1	2	3	1	2	3	1	2	3	1	2	3	1	2	3	1	2	3	1	2	3	1	2	3	1	2	3	1	2	3	1	2	3	1	2	3
I	97	100		53	100	√	35	100	√	97	100		87	100		22	88		0.09	61		32	61		21	61		0.06	55		29	55		0.04	16		27	0		83	94	
II	100	100		95	100		23	88		100	100		100	100		38	83	√	19	77	√	9	61	√	9	55		9	55		28	0	√	42	11		9	11		28	16	√
III	86	94		86	94		0	0		86	100		86	77		86	100		86	100		86	100		86	100		81	100		81	88		13	0		77	72		0	0	
IV	96	100		92	100		55	0		96	86		88	94		77	55		3	55		11	50	√	0	0		0	0		44	61		40	61		40	61		29	38	
V	90	100		90	88		0	0		90	88		90	88		90	88		0	0		0	0		0	0		0	0		0	0		0	0		0	0		0	0	
VI	100	100		56	100		12	50		100	100		97	100		7	5		0	0		5	5		2	5		0	0		30	100		35	0		5	24		43	22	
IX	92	94		60	94	√	20	94	√	67	100	√	62	94		60	88	√	5	88	√	5	88	√	2	88	√	0	0		30	94	√	32	22	√	42	0	√	37	33	
X	97	100		34	100	√	29	55	√	94	88		90	88		33	55		16	16		28	22	√	18	22		6	16		6	0		5	0		1	16		77	55	√
XI	96	100		22	100		24	50	√	94	100		86	83		21	55		10	50		20	50	√	14	50		7	0		3	22	√	4	22	√	1	22	√	10	55	
XII	72	88	√	77	88	√	0	0		77	94		77	83	√	0	83		0	0		10	38	√	10	10		0	0		0	0		4	4		4	4		10	33	
XIII	100	94		95	94		18	27		100	88		100	88		0	0		0	0		0	0		0	0		0	0		68	33		100	88		0	0		9	55	
XIV	90	100		47	50		32	37	√	85	83	√	80	55	√	37	50	√	5	0		37	0	√	37	0	√	22	0		7	27		80	94		84	83	√	28	44	
XVI	100	100		57	80		58	0		100	100		100	10	√	23	80		76	50		14	10		14	12		0.04	0		0.04	0		0.04	95	√	0.04	0		0.04	0	
XVII	100	88		100	88		81	88		100	83		100	83		81	100		32	83	√	8	22	√	8	22	√	4	22		84	88		81	94		84	83		28	44	
XVIII	95	100		68	100	√	35	100		95	100		11	77		30	66	√	0	0		15	55	√	15	61	√	3	55		16	77		11	55		13	72	√	76	72	
XIX	86	94		81	94		0	0		86	94		93	94		6	16		0	0		0	0		0	0		0	0		0	0		0	0		0	0		26	0	
XXI	90	100		90	88		90	88		90	88		90	88		0	0		0	0		0	0		0	0		0	0		0	0		0	0		0	0		0	0	

*Note*: The numbers inside the table are percentages. 1: Percentages phase1, 2: Percentages phase2, 3: √ the phase3 confirms the experts.

Abbreviations: BC, blood culture; Hb, hemoglobin; HCT, hematocrit; INR, international normalized ratio; MCH, mean corpuscular of hemoglobin; MCHC, mean corpuscular hemoglobin concentration; MCV, mean corpuscular volume; PLT, platelet; PT, prothrombin time; PTT, partial thromboplastin time; RBC, red blood cell; WBC, white blood cell; WBC/LY, white blood cell/lymphoma; WBC/other, white blood cell/other.

A total of 187 data elements (11 unique laboratory tests * 17 chapters of ICD10 = 187 data) for blood biochemistry tests were reported in the summary sheets. These were reported with respect to the diagnosis classified under ICD‐10 categories (see Table 2). Of these data, 68 elements were approved in two phases (specialists' survey and expert panel) and were considered essential. Among data reported for these tests, there were 97 data elements that were reported in less than 20% of sheets, which were excluded from the study. The remaining 24 elements were reviewed by the experts' panel and 10 elements were verified. At last, a total of 77 data elements of blood biochemistry tests were included in the MDS.

**Table 2:** The frequency of blood chemistry tests reported in patient summary sheets, verified by physicians and included in MDS.

11 data	BS			Cr			BUN			Na			K			Ca			ALB			CHOL			Trig			Bili. T			Bili. D		
ICD10 chapter	1	2	3	1	2	3	1	2	3	1	2	3	1	2	3	1	2	3	1	2	3	1	2	3	1	2	3	1	2	3	1	2	3
I	48	100	√	87	100		88	100		86	100		88	100		17	55		14	0		0.1	0		0.1	0		22	27	√	22	27	√
II	85	100		100	100		100	100		100	100		100	100		7	0		4	0		0	0		0	0		9	0		9	0	
III	81	77		86	100		86	100		86	100		86	100		0	0		0	0		0	0		0	0		0	0		0	0	
IV	96	100	√	56	100		12	0		96	100		92	100		66	44		0	0		0	0		0	0		0	0		0	0	
V	0	0		94	88		94	88		94	88		94	88		0	0		0	0		0	0		0	0		0	0		0	0	
VI	56	83		97	100		100	100		97	100		97	100		76	0		0	0		0	0		0	0		0	0		0	0	
IX	77	88	√	87	94		85	94		82	94		82	94		0	0		0	0		0	0		0	0		0	0		0	0	
X	61	88		93	88		89	88		92	88		91	88		17	27		0	0		0	0		0	0		0	0		0	0	
XI	60	83	√	91	88		91	88		90	88		90	88		17	0		0	0		0	0		0	0		0	0		0	0	
XII	68	83		72	88	√	72	88	√	72	88	√	50	88	√	0	0		0	0		0	0		0	0		0	0		0	0	
XIII	100	94		100	88		100	88		95	88		95	88		0	0		0	0		0	0		0	0		0	0		0	0	
XIV	90	100		80	88		80	88		80	88		80	88		17	50		5	44		0	0		0	0		5	0		0	0	
XVI	95	92		95	100		95	100		95	100		95	100		45	70		0	0		0	0		0	0		0	10		0	10	
XVII	100	83		92	94		88	94		92	94		92	94		84	83		0	0		0	0		0	0		0	0		0	0	
XVIII	95	100		91	100		91	100		90	100		90	100		23	55		0	0		0	0		0	0		0	0		0	0	
XIX	66	77		81	94		81	94		81	94		81	94		26	50		13	0		0	0		0	0		13	0		13	0	
XXI	7	0		7	0		7	0		7	0		7	0		0	0		0	0		0	0		0	0		0	0		0	0	

*Note*: The numbers inside the table are percentages. 1: Percentages phase1, 2: Percentages phase2, 3: √ the phase3 confirms the experts.

Abbreviations: ALB, albumin; Bili. D, direct bilirubin; Bili. T, bilirubin total; BS, blood sugar; BUN, blood urea nitrogen; Ca, calcium; CHOL, cholesterol; Cr, creatinine; K, potassium; Na, sodium; Trig, triglyceride.

A total of 102 enzyme and hormone tests (6 unique laboratory tests * Chapter 17 ICD10 = 102 data) were reported in the patient's summary sheets. Nine of these data elements were reported in more than 80% of summary sheets. As a result, they were considered essential data elements and were included in the MDS. However, there were 62 data elements that were identified in less than 20% of summary sheets and less than 20% of physicians that were contacted were in agreement with their exclusion in the final list of the MDS. Of the 31 remaining elements, 14 were confirmed in the experts' panel. Eventually, a total of 23 data elements were included in the MDS for enzyme and hormone tests. In this case, there were six unique data elements (see Table 3).

**Table 3:** The frequency of enzymes and hormone tests reported in patient summary sheets, verified by physicians and included in MDS.

6 data	ALT			ALK‐P			AST			LDH				T4			TSH		
ICD10 chapter	1	2	3	1	2	3	1	2	3		1	2	3	1	2	3	1	2	3
I	35	22	√	35	22	√	35	22	√		0	0		0.1	5		0.1	5	
II	100	88		100	88		100	88			100	88		100	88		100	88	
III	22	0		22	0		22	0	√		0	0	√	0	0		0	0	
IV	51	66		51	66		51	66			0	0		18	100		18	100	√
V	60	83		60	83		60	83			0	0		40	0		40	0	√
VI	28	27		28	27		28	27			0	0		0	0		0	0	
IX	45	27	√	45	27	√	45	27	√		0	0		12	0		0	0	
X	0	0		0	0		0	0			0	0		2	0		0	0	
XI	14	88		14	88		14	88			0	0		0	0		0	0	
XII	45	72	√	45	72	√	45	72	√		0	0		0	0		0	0	
XIII	0	0		0	0		0	0			0	0		0	0		0	0	
XIV	20	11		20	11		20	11			0	0		0	0		0	0	
XVI	0.04	15		0.04	15		0.04	15						0	5		0	5	
XVII	84	88		84	88		84	88			0	0		0	0		0	0	
XVIII	23	66	√	23	66	√	23	66	√		0	0		0	0		0	0	
XIX	0	0		0	0		0	0			0	0		0	0		0	0	
XXI	0	0		0	0		0	0			0	0		0	0		0	0	

*Note*: The numbers inside the table are percentages. 1: Percentages phase1, 2: Percentages phase2, 3: √ the phase3 confirms the experts.

Abbreviations: ALK‐P, alkaline phosphatase; ALT, alanine aminotransferase; AST, aspartate aminotransferase; LDH, lactate dehydrogenase; T4, thyroxin; TSH, thyroid stimulating hormone.

Of the 153 data elements (9 unique laboratory tests * 17 chapter ICD10 = 153 data) for urine and stool analysis, no data elements were reported in more than 80% of the summary sheets. Moreover, due to the low report, 52 elements were removed from the study. Of the 101 elements evaluated in the experts' panel, 62 were approved as the required data and were included in the MDS. Among these data, there were nine unique data elements (see Table 4).

**Table 4:** The frequency of urinalysis and stool tests reported in patient summary sheets, verified by physicians and included in MDS.

9 data	UA			UA/RBC			UA/WBC			UA/SPG			UC			SE			SE/RBC			SE/WBC			OB		
ICD10 chapter	1	2	3	1	2	3	1	2	3	1	2	3	1	2	3	1	2	3	1	2	3	1	2	3	1	2	3
I	61	94	√	53	55	√	61	55	√	61	55	√	72	50	√	79	55	√	77	55	√	77	55	√	72	94	
II	52	83		9	0		4	0		0	0		4	0		52	83		9	0		9	0		9	0	
III	27	44		0	0		0	0		0	0		0	0		54	55		0	0		0	0		0	0	
IV	62	38	√	29	38	√	29	38	√	29	38	√	66	38	√	66	55		33	38		0	0		25	38	
V	0	0		0	0		0	0		0	0		0	0		0	0		0	0		0	0		0	0	
VI	35	55		5	0		35	0		35	0		43	0		69	50		48	0		48	0		46	0	
IX	57	44	√	35	44	√	35	44	√	35	44	√	57	44	√	57	44	√	37	44	√	37	44	√	40	55	
X	72	44	√	72	33	√	53	38		68	38		70	38		77	50		80	0		80	0		82	0	
XI	67	72	√	71	55	√	71	55	√	74	55	√	74	55	√	66	94	√	64	94	√	64	94	√	68	94	
XII	36	27		31	0		27	0		22	0		8	0		9	33		9	0		9	0		9	0	
XIII	36	50		9	0		31	0		31	0		0	0		9	66		9	0		9	0		9	0	
XIV	72	100	√	40	100	√	42	100	√	47	100	√	67	100	√	77	38	√	72	0	√	0	0	√	67	0	√
XVI	28	7		14	0		0.09	0		0.09	0		0.09	10		0.09	10		0.09	0		0.04	0		0.04	0	
XVII	40	88	√	60	27	√	60	27	√	56	27	√	28	27	√	52	88		52	44		52	44		20	38	
XVIII	66	83	√	76	38	√	76	38	√	78	38	√	75	44	√	75	88	√	75	55	√	75	55	√	75	61	√
XIX	26	83	√	26	0	√	26	0	√	26	0	√	26	0	√	33	77	√	26	0		26	0		26	0	√
XXI	0	0		0	0		0	0		0	0		0	0		0	0		0	0		0	0		0	0	

*Note*: The numbers inside the table are percentages. 1: Percentages phase1, 2: Percentages phase2, 3: √ the phase3 confirms the experts.

Abbreviations: OB, occult blood; SE, stool examination; SE/RBC, stool examination/red blood cell; SE/WBC, stool examination/white blood cell; UA, urinalysis; UA/RBC, urinalysis/red blood cell; UA/SPG, urinalysis/gravity specific; UA/WBC, urinalysis/white blood cell; UC, urine culture.

There were 119 data elements regarding other laboratory tests that were extracted from the summary sheets, (7 unique laboratory tests * 17 chapter ICD10 = 119 data). Among them none were reported in more than 80% of the summary sheets. Of these data, 92 were reported in less than 20% of the sheets and were thus excluded from the final list. In the experts' panel, 27 elements of these laboratory data were reviewed and the experts were in agremment of inclusion of 14 elements in the MDS. Among these data, there were two unique data elements (see Table 5).

**Table 5:** The frequency of other tests reported in patient summary sheets, verified by physicians and included in MDS.

7 data	ESR			CRP			Pco_2_			Hco_3_			SaO_2_			P			CSF		
ICD10 chapter	1	2	3	1	2	3	1	2	3	1	2	3	1	2	3	1	2	3	1	2	3
I	72	88	√	71	88	√	0.04	5		0	0		0	0		0	0		0	0	
II	14	88	√	14	88	√	0	0		0	0		0	0		14	0		4	0	
III	54	50		2	55		0	0		0	0		0	0		0	0		0	0	
IV	59	55		66	55		3	0		0	0		0	0		0	0		0	0	
V	0	0		0	0		0	0		0	0		0	0		0	0		0	0	
VI	16	0		27	0		10	0		0	0		0	0		0	0		5	10	
IX	37	44	√	42	50	√	0	0		0	0		0	0		0	0		0	0	
X	61	50		61	45		13	0		0	0		0	0		2	0		0	0	
XI	73	88	√	63	55	√	5	0		0	0		0	0		0	0		0	0	
XII	68	88		63	55		0	0		0	0		0	0		0	0		0	0	
XIII	36	94	√	36	94	√	0	0		0	0		0	0		0	0		0	0	
XIV	60	45	√	65	45	√	10	0		5	0		10	0		2	0		0	0	
XVI	0.09	0.09		0.04	0		0	0		0	0		0	0		0	0		0	14	
XVII	28	55		28	50		0	0		0	0		0	0		0	0		0	0	
XVIII	76	83	√	75	83	√	5	0		1	0		1	0		0	0		0	0	
XIX	6	72		26	77		0	0		0	0		0	0		0	0		0	0	
XXI	0	0		0	0		0	0		0	0		0	0		0	0		0	0	

*Note*: The numbers inside the table are percentages. 1: Percentages phase1, 2: Percentages phase2, 3: √ the phase3 confirms the experts.

Abbreviations: CRP, C‐reactive protein; CSF, cerebrospinal fluid; ESR, erythrocyte sedimentation rate; Hco_3_, oxygen saturation; P, phosphate; Pco_2_, bicarbonate; SaO_2_, oxygen saturation.

In the second phase of the study, 18 data elements were suggested by physicians to be included in the MDS. Even though, the physicians sugested to include these additional data elements in the MDS, they were not verified by experts in the third phase and, as a result, were not added to the final MDS list. For two chapters of eye and ear diseases, experts in the third phase decided the most commonly used tests in other chapters should be considered as MDS for these two chapters (Supporting Information: Appendix 3).

## 4 DISCUSSION

A MDS of laboratory data of summary sheets was developed based on the data extracted from patients' records, physicians' comments, and expert opinions. Reviewing laboratory data and classifying them based on coded diagnoses showed that registering most laboratory data follow a predictable pattern based onn the diagnosis codes. Identifying these patterns will be useful for the development of an automated system that canelectronically extract the most relevant laboratory for summary sheets from medical records. A total of 292 data elements were determined for the MDS of summary sheet of pediatric patients. When developing this MDS, the laboratory tests were chosen based on the diagnostic codes categories from ICD‐10 chapters (Supporting Information: See Appendix 3). Therefore, this methods will enable hospitals to update their information systems in such way that once a patient's diagnosis code is entered based on the ICD‐10 classification, the laboratory data for that diagnosis will be automatically added to the patient's summary sheet.^18^ This capability not only expedites documentation but also improves the quality and accuracy of records. Since coding diagnoses based on ICD‐10 is mandatory in Iran and many other countries,^19^ designing a MDS with regard to diagnostic codes is not only feasible but also of great use.^18^

The most common laboratory data included in the MDS were related to hematology tests. More than half of the hematology tests reported in the patient's summary sheet were ultimately approved and included in the MDS. In the first phase of the study, the data elements of WBC, WBC/LY, Hb, and PLT related to hematology tests and Cr, BUN, Na and K related to blood biochemistry tests were documented in more than 80% of summary sheets. The panel of experts agreed that hematology tests should be recorded regularly in the summary sheets of pediatric patients for diagnosing most diseases.

In the first phase of the study, enzyme and hormone tests as well as other tests were reported in less than 80% of summary sheets and were approved by less than 80% of physicians in the second phase. In the third phase, only a few of these tests (14 data elements) were approved and added to the MDS of laboratory data. For certain diagnoses such as neoplasms and digestive diseases, more specialized tests such as ALT‐ALK‐P, AST, LDH, ESR, UA, and SE were included in the MDS to be registered in the summary sheets. The data incorporated in the MDS can be recorded automatically in the summary sheet from the hospital information system. If necessary, physicians can record, based on the patient's diagnosis, the necessary tests that are not part of the proposed MDS.

For their daily work, physicians need to access a summary of clinical data. The problem of large amount of data has always been a challenge when exchanging data between hospitals and care providers, which has heightened the need for a tool to summarize such data.^20^ Designing an MDS of clinical data is essential to facilitating the exchange of electronic health data between different systems.^21^ However, despite this functionality, no study has been so far conducted on the development of an MDS for laboratory data to be integrated in the patient summary sheet.

In the clinical field, this tool could be developed in a variety of ways. Ringer and colleagues designed a nursing MDS by examining different minimum data sets, and combining these data sets. in a panel of experts, they developed a final list of these data for Australia.^22^ Kelimstra and colleagues introduced an MDS for the pathology report of patients with neurological and endocrine diseases in the United States. In this study, the first draft of an MDS was established for the pathology report based on ICD‐10 codes. Then, a medical team of different specialists approved the initial draft of MDS by the Delphi methodology by answering yes and no questions for each data item. Data that verified by more than 80% of physicians were added to the MDS and others were discussed and confirmed during a focus group meeting.^23^

Ahmadian and colleagues suggested a national data set for preoperative assessment in the Netherlands, with the aim of interacting and exchanging data between different electronic health records and better communication between care providers. First, they systematically reviewed the literature to retrieve the data elements that are important in the assessment of patients before surgery. The retrieved data were discussed in eight focus group meetings. Accordingly, the national preoperative data set was developed. In this data set, 82 elements were designed by experts, accounting for 76% of the data elements extracted from previous studies.^24^

In the study of Fallahnejad et al.^25^ a minimum data set for electronic documentation of progress note in the ICU was developed through literature review, and group discussion.

Moeil Tabaghdeh et al.^26^ developed the minimum data set of thalassemia patients in the electronic health record based on two stages. In the first stage, data elements were extracted from the medical record, and in the second stage, the data was validated by experts using the Delphi technique. The data elements proposed in this study can be considered as a suitable data set for inclusion in manual systems and electronic medical records, and can be used as a national document based on the needs of patients.

The method we used in this study is a mixed one which is based on reviewing patients' records, surveying care providers, and consulting expert opinions. The advantage of this study, compared to others, is in reviewing medical records, which led to developing an MDS that is closer to the current needs of users.

Designing an MDS of laboratory tests for the summary sheets of pediatric patients and implementing it in hospital information systems can help to automatically record these data in patients' summary sheets and save physicians' time. This can facilitate the discharge process and improve physicians' performance in managing patients. Moreover, as the application of this MDS is easy and in line with the workflow of physicians, its acceptance rate will be high.^27^

The strengths of this study include a large number of reviewed summary sheets and the continuation of the sampling process to achieve data saturation. Another is the categorization of tests based on the encoded diagnoses provided through the ICD‐10 classification. The method used in this study can also be employed to design a laboratory MDS for summary sheets of other hospital wards. The results can help physicians to better record laboratory data in a summary sheet and enable automatic registration of these data.

One of the limitations of the study concerns the illegibility of paper‐based summary sheets due to the sloppy handwriting of physicians. To tackle this problem, hospital nurses and medical coders who had experience with the writing style of the physicians were asked to help the researcher in understanding the summary sheets. Another limitation was the lack of cooperation of physicians in the second phase of the study. To overcome this limitation, the Vice‐Chancellor of Zahedan University of Medical Sciences formally invited physicians to cooperate and collaborate in this research.

## 5 CONCLUSIONS

The design of the minimum data set of laboratory data for the summary sheet of patient records will facilitate the exchange of information in the electronic health record. A minimum data set will increase the accuracy and speed of recording data in an ESS and will facilitate the provision of services to patients by the medical staff. The MDS developed in this study is linked to diagnosis codes and determining the category of diagnosis. It makes possible specifying which laboratory data element should be recorded in the patient summary sheet. The method proposed in this study enhances summarization and automation of ESSs. It could also be deployed to design an MDS for most clinical data recorded in ESSs. Since the ICD system is a global standard for classifying information recorded in patients' records, it is suggested to design minimum clinical data sets by considering this classification system when it is feasible.

## AUTHOR CONTRIBUTIONS

**Farzaneh Shahbakhsh**: Conceptualization; data curation; investigation; writing—original draft; writing—review & editing. **Reza Khajouei**: Conceptualization; formal analysis; investigation; methodology; writing—original draft; writing—review & editing. **Azam Sabahi**: Conceptualization; methodology; writing—original draft; writing—review & editing. **Yousef Mehdipour**: Conceptualization; formal analysis; writing—original draft; writing—review & editing. **Leila Ahmadian**: Conceptualization; formal analysis; investigation; methodology; project administration; supervision; validation; writing—original draft; writing—review & editing.

## CONFLICT OF INTEREST STATEMENT

The authors declare no conflict of interest.

## ETHICS STATEMENT

This study was approved by the Research Ethics Committee of Kerman University of Medical Sciences (No.: IR.KMU.REC.1396.1345).

## TRANSPARENCY STATEMENT

The lead author Leila Ahmadian affirms that this manuscript is an honest, accurate, and transparent account of the study being reported; that no important aspects of the study have been omitted; and that any discrepancies from the study as planned (and, if relevant, registered) have been explained.

## Supporting information

Supporting information.

## Data Availability

Further data is available from the corresponding author on reasonable request. She (Leila Ahmadian) affirms that this manuscript is an honest and accurate study, and present unbised results. The authors confirm that the data supporting the findings of this study are available within the article (and/or) its Supporting Information.
