# Supplementary material for: Designing a minimum data set of laboratory data for the electronic summary sheet of pediatric ward in Iran: A cross‐sectional study
Source: Health Sci Rep. 2023 Jun 7;6(6):e1315. doi: 10.1002/hsr2.1315 (PMC10248033; doi:10.1002/hsr2.1315)
Supplement: Supplementary file 1 — Supporting information. [file HSR2-6-e1315-s001.docx]

| **Appendix 1: International Statistical Classification of Diseases and Related Health Problems** | | |
| --- | --- | --- |
| ***Chapter*** | **Title** | **Frequency of Record** |
| ***I*** | Certain infectious and parasitic diseases | 62 |
| ***II*** | Neoplasms | 21 |
| ***III*** | Diseases of the blood and blood-forming organs and certain disorders involving the immune mechanism | 22 |
| ***IV*** | Endocrine, nutritional and metabolic diseases | 27 |
| ***V*** | Mental and behavioral disorders | 10 |
| ***VI*** | Diseases of the nervous system | The record did not exist |
| ***VII*** | Diseases of the eye and adnexa | The record did not exist |
| ***VIII*** | Diseases of the ear and mastoid process | 39 |
| ***IX*** | Diseases of the circulatory system | 40 |
| ***X*** | Diseases of the respiratory system | 75 |
| ***XI*** | Diseases of the digestive system | 90 |
| ***XII*** | Diseases of the skin and subcutaneous tissue | 22 |
| ***XIII*** | Diseases of the musculoskeletal system and connective tissue | 22 |
| ***XIV*** | Diseases of the genitourinary system | Not applied |
| ***XV*** | Pregnancy, childbirth and the puerperium | 40 |
| ***XVI*** | Certain conditions originating in the perinatal period | 16 |
| ***XVII*** | Congenital malformations, deformations and chromosomal abnormalities | 25 |
| ***XVIII*** | Symptoms, signs and abnormal clinical and laboratory findings, not elsewhere classified | 60 |
| ***XIX*** | Injury, poisoning and certain other consequences of external causes | 15 |
| ***XX*** | External causes of morbidity and mortality | Not applied |
| ***XXI*** | Factors influencing health status and contact with health services | 13 |


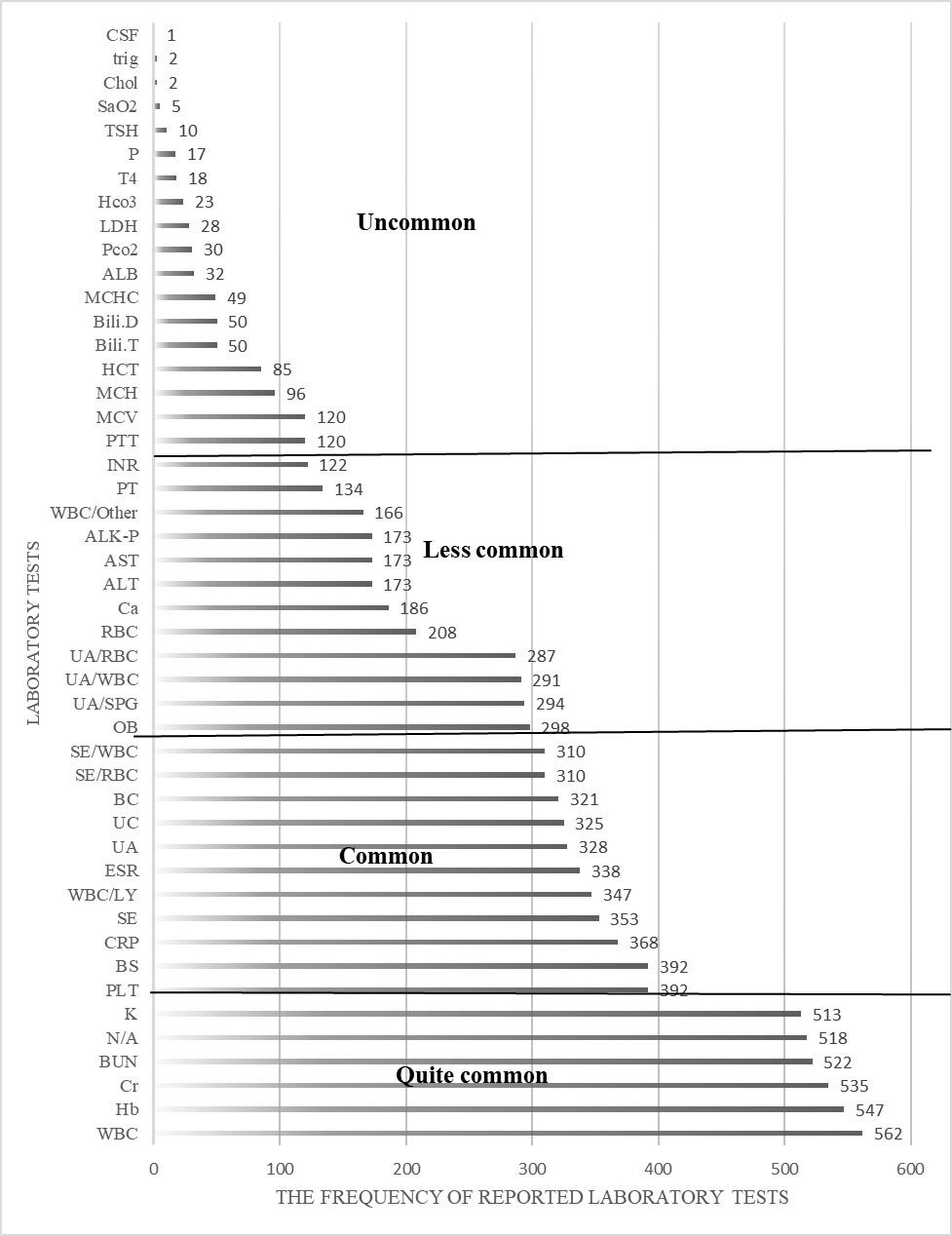


**Figure 1: The Frequency of Laboratory Data Reported in Summary Sheets**

**Appendix 2**

**Appendix 3**

**Minimum data set of laboratory based on the ICD10 chapters**

| *I. Certain infectious and parasitic diseases* |
| --- |
| *WBC,WBC/Other, WBC/LY, Hb, BS, PLT, Cr, BUN, Na ,K ,ALT, ALK-P, AST, UA,UC,SE ,CRP,ESR,UA/RBC,UA/WBC, UA/SPG, BC, SE/RBC, SE/WBC, Bili_T, Bili_D, BC* |

| *II. Neoplasms* |
| --- |
| *WBC,* *WBC/LY ,Hb, PLT, RBC, HCT, MCV, BC, Cr, BUN, Na, K, ALT, ALK-P, AST, LDH, PT ,CRP, ESR, PT, BS, T4, TSH* |

| *III. Diseases of the blood and blood-forming organs and*  *certain disorders involving the immune mechanism* |
| --- |
| *WBC,WBC/LY, HB, Cr, BUN, Na, K, RBC, HCT, MCV, MCH, MCHC, PT, AST, LDH, PLT* |

| *IV. Endocrine, nutritional and metabolic diseases* |
| --- |
| *WBC, WBC/LY, HB, BS, PLT, Na, K, MCV, TSH, UA, UC, UA/RBC,UA/WBC,UA/SPG* |

| *V. Mental and behavioral disorders* |
| --- |
| *WBC, WBC/LY, HB, BS, PLT, RBC, Cr, BUN, Na ,K, TSH* |

| *VI. Diseases of the nervous system* |
| --- |
| *WBC,WBC/LY, Hb, BS, PLT, Cr, BUN, Na, K, ALT, ALK-P, AST* |

| *VII. Diseases of the eye and adnexa* |
| --- |
| *WBC, Hb, PLT, Cr, BUN, Na, K* |

| *VIII. Diseases of the ear and mastoid process* |
| --- |
| *WBC, Hb, PLT, Cr, BUN, Na, K* |

| *IX .Diseases of the circulatory system* |
| --- |
| *WBC,WBC/Other, WBC/LY, Hb, BS, PLT, Cr, BUN, Na, K ,RBC,HCT,MCV,PTT,ALT,ALK-P,AST,PT,INR, UA,UC,SE,CRP,ESR,UA/RBC,UA/WBC,UA/SPG, SE/RBC,SE/WBC,MCH* |

| *X. Diseases of the respiratory system* |
| --- |
| *WBC,WBC/Other, WBC/LY, Hb, PLT, Cr, BUN, Na, K,MCV, UA, UA/RBC, BC* |

| *XI. Diseases of the digestive system* |
| --- |
| *WBC, WBC/Other, Hb, PLT, Cr, BUN, Na, K, PT, PTT, INR, MCV, UA, UC, SE,CRP, ESR,UA/RBC,UA/WBC,UA/SPG, SE/RBC,SE/WBC,BS* |

| *XII .Diseases of the skin and subcutaneous tissue* |
| --- |
| *WBC/Other, WBC/LY, PLT, MCV, Cr, BUN, Na, K, ALT, ALK-P, AST* |

| *XIII .Diseases of the musculoskeletal system and connective tissue* |
| --- |
| *WBC, WBC/LY, Hb, BS, PTT, Na, K, PLT ,Cr, BUN* |

| *XIV.Diseases of the genitourinary system* |
| --- |
| *WBC, WBC/Other, Hb, BS, PLT, Cr, BUN, Na, K,RBC,MCV,MCH,PTT, INR, UA,UC,SE,CRP,ESR,UA/RBC,UA/WBC,UA/SPG ,SE/RBC, ,OB* |

| *XVI. Certain conditions originating in the perinatal period* |
| --- |
| *WBC, PLT, Hb, Cr, BUN, K, Na, BS, PTT, INR* |

| *XVII. Congenital malformations, deformations and Chromosomal abnormalities* |
| --- |
| *WBC,WBC/LY,Hb,PLT,Cr,BUN,Na,K,RBC,HCT,MCV,MCH,PT,INR,BS, ALT, ALK-P, AST, PCO2, UA, UC, CRP, ESR,UA/RBC,UA/WBC,UA/SPG,PTT* |

| *XVIII. Symptoms, signs and abnormal clinical and laboratory findings not elsewhere classified* |
| --- |
| *WBC,WBC/LY, Hb, PLT, Cr, BUN, Na, K, ALT,ALK-P,AST,PTT, UA,UC,SE,UA/RBC,UA/WBC,UA/SPG, OB,BS,SE/RBC, SE/WBC, OB* |

| *XIX .Injury, poisoning and certain other consequences of external causes* |
| --- |
| *WBC, WBC/LY, Hb, PLT, Cr, BUN, Na, K, SE, UA, UA/RBC, UA/WBC, UA/SPG* |

| *XXI .* Factors influencing health status and contact with health services |
| --- |
| *WBC, WBC/LY,WBC/other, Hb, PLT* |
